# Triazole Fungicides Inhibit Zebrafish Hatching by Blocking the Secretory Function of Hatching Gland Cells

**DOI:** 10.3390/ijms18040710

**Published:** 2017-04-04

**Authors:** Javiera F. De la Paz, Natalia Beiza, Susana Paredes-Zúñiga, Misque S. Hoare, Miguel L. Allende

**Affiliations:** 1 Center for Genome Regulation, Facultad de Ciencias, Universidad de Chile, Santiago 8370415, Chile; nbbeiza@gmail.com (N.B.); sparedesz@ug.uchile.cl (S.P.-Z.); mallende@u.uchile.cl (M.L.A.); 2 Corporación para el Desarrollo de las Ciencias Ambientales, CODECIAM, Santiago 8270966, Chile; sol.misque@gmail.com

**Keywords:** zebrafish, triazoles, fungicides, triadimefon, hatching, choriolysin, Hatching Enzyme 1, bioassay, dopamine

## Abstract

In animals, hatching represents the transition point from a developing embryo to a free-living individual, the larva. This process is finely regulated by many endogenous and environmental factors and has been shown to be sensitive to a variety of chemical agents. It is commonly evaluated in bioassays in order to establish the effects of different agents on early development and reproductive capabilities in fish and other aquatic animals. In fish, the breakdown of the chorion is achieved by the secretion of choriolysin by hatching gland cells (HGCs) into the perivitelline space (PVS), coupled with spontaneous movements of the developing larva. In this work, we used zebrafish to assay the effects of a family of widely used agrochemicals—triazoles Triadimefon (FON), Triadimenol (NOL) and free triazole (1,2,4-T)—on hatching success. We found a strong inhibition of hatching by triazole exposure which was correlated with morphological changes and a reduction in the secretory function of the HGCs. As a consequence, the release of choriolytic enzymes by HGCs was reduced. We also found that HGC secretion reduction after exposure to FON can be rescued by co-incubation with a dopamine D2 receptor antagonist but not by antagonists of the D1-like receptors. This suggests a specific pathway through which this family of fungicides may be impairing a critical event in the fish life cycle.

## 1. Introduction

### 1.1. Environmental Disruption of Hatching

Hatching is a critical stage in the life cycle of metazoans. It corresponds to the release of individuals from the egg envelope or chorion and it marks the end of embryogenesis and the beginning of the larval stage or birth [1]. Hatching in fish occurs when developing organisms are still consuming maternally supplied nutrients but, at the same time, their developing nervous system, organs, and muscles allow them to search for their own food [2]. On the other hand, hatched animals are more vulnerable to predatory attacks, mechanical and osmotic stress, and chemical compounds present in the environment, making timing alterations in this process potentially lethal.

Hatching alterations (retardation, inhibition, or acceleration) in fish can be caused by many different endogenous and exogenous factors including environmental pollutants like hexachlorobenzene (HCB), 2.3,7,8-tetrachlorodibenzo-p-dioxin (TCDD), benzene, decabrominated diphenyl ether (BDE-209), oxybenzone, and heavy metals, among other multiple organic compounds [3,4,5,6,7,8]. Furthermore, effects have been reported for oxygen availability (in *F. heteroclitus* an O_2_ concentration over 4–6 mg/L inhibits, and below 1 mg/L stimulates hatching) [9,10], Central Nervous System (CNS) chemical modulators such as MS-222 and dopamine (DA) receptor agonists and antagonists, and for hormones like cathecolamines and prolactin [11,12]. This detrimental response is generally classified as a sub-lethal effect on toxicity bioassays, even when it can result in death if—in the short term—it is not reversed. Since hatching is a critical step in reproduction, its disruption can cause a negative impact at the population level, affecting natural and artificial ecosystems. In contrast, it may also be a useful tool for phytosanitary and clinical applications. For instance, in the case of parasitic diseases, it can represent an alternative for controlling pest proliferation [13,14]. For the aquaculture industry, reduced hatching success of fish represents a serious problem by reducing the viability of the eggs [15] with the ensuing economic impact. In the literature, there is scant data on the cellular and molecular mechanisms related to hatching inhibition exerted by environmental toxicants such as pesticides or heavy metals, which can easily reach surface and ground waters, affecting aquatic organisms including fish.

### 1.2. Hatching Onset Regulation in Fish

Hatching in fish is regulated, on the one hand, by endogenous factors such muscle contractions, release of proteolytic enzymes from specialized cells, and hormonal levels. On the other hand, exogenous factors may also play a role, including light-dark cycles and water oxygen levels [9,16,17]. Due to its sensitivity to environmental conditions, hatching success is commonly used for assessing the effects of chemical agents on early development and reproduction [3,6,18,19,20,21,22]. Many studies have described that specific pollutants and physical agents alter the hatching of fish and other aquatic animals [23,24,25,26], but the mechanisms and pathways involved remain poorly understood.

Before hatching, the fish embryo releases a mixture of proteases—commonly called choriolysins—into the perivitelline space (PVS), in order to digest the chorion [2]. In the particular case of zebrafish, a single enzyme is responsible for breaking down this protein barrier: Hatching Enzyme 1 (HE1) [27]. The weakening of the chorion allows the first spontaneous movements of the larvae to tear it apart, setting them free [2]. These events take place, in the case of zebrafish, between 48 and 72 h post-fertilization (hpf) at 25–28 °C [28]. In many teleosts and amphibians, there is a transient group of cells called hatching gland cells (HGCs) that produce, accumulate, and secrete choriolysins that will digest glycoproteins present on the internal side of the chorion, the internal zona radiata [2,29]. In zebrafish, these cells are individual glands organized as an epithelial monolayer that become differentiated and mature on the surface of the pericardial region of the yolk at two days post-fertilization (dpf). They have been proposed to be specified during gastrulation, differentiating in the pre-hatching period, a stage at which they show a highly refractive cytoplasm replete with granules. Prior to the onset of hatching, granules are secreted by an unknown mechanism [28,30]. It has been suggested that prolactin (PRL) could act as a neuroendocrine signal that stimulates the release of hatching enzymes since it has been shown that this hormone induces HGC secretion ex vivo [12]. Moreover, other studies have shown that the neurotransmitter dopamine (DA), a well-known PRL release inhibitor, can block this secretory function, while DA antagonists can reverse the effect [11].

### 1.3. Triazole Fungicide Toxicity and Environmental Relevance

Triazole fungicides (Triadimefon, Triadimenol, and 1,2,4-T) are considered as potential water pollutants. Triadimefon (FON) and Triadimenol (NOL) are specified as a threat to terrestrial and aquatic animals due to their potential for dispersion in the environment [31,32]. A field residue measurement of FON and its first degradation product, NOL—which retains its antifungal activity—on land runoff from golf course fairways treated with FON to control fungal disease, detected concentrations of up to 0.96 mg/L of FON and 0.15 mg/L of NOL [33], demonstrating the risk of their dispersion into the environment.

Triazole derivatives are commonly used for the treatment and prevention of fungal infections not only in agriculture but also in medicine, since they inhibit the formation of ergosterol, an important component of the fungal cell wall [34]. Unfortunately, in mammals, they also impair steroidogenesis therefore affecting reproduction and development [35] and there is substantial literature on the impacts of FON exposure to the endocrine system of mammals [36,37,38,39] and fish [32,40,41,42]. Additional work has described the teratogenic effects induced by FON and NOL exposure in ascidians [43], amphibians [44], and murine mammals [45]. The teratogenic effects of triazoles have been linked to alterations of retinoid acid (RA) metabolism and signaling, which may interfere with normal migration of the neural crest cells [46]. In zebrafish, the effects of FON, include reduction of growth, breeding success, and egg viability; teratogenesis; edema; and a decrease in hatching rates [32,40,41,47].

In humans, the TOXNET database contains reports of accidental overexposures to FON in its commercial form, Bayleton 50%, in which exposed persons reported, among many other symptoms, hyperactivity [48], an effect that has also been reported in rats [49,50]. Further, FON is known to inhibit DA reuptake in mammals by blocking the action of the dopamine transporter, resulting in increased extracellular levels of DA and its neurotransmission [51]. Interestingly, in the hypophysis, DA acts as an inhibitor of PRL release by coupling to D2-like receptors in lactotroph cells [52,53].

### 1.4. Some Advantages of Zebrafish in Toxicology Research

Many fish species are recommended for standard analysis of chemical pollutants or environmental samples [20,21,22]. In particular, the zebrafish has been intensively used in ecotoxicological studies as it has unique characteristics that make it an attractive model organism. Adults are small (5 cm), spawn continuously throughout the year, display a short life cycle (two to three months), embryos develop rapidly (48 h at 28 °C), and the embryos and chorion are optically transparent. Further, they are easy to breed in the laboratory under standard conditions [54] and its genome contains a large proportion of genes that have orthologs in mammals [55]. All of this makes zebrafish an ideal model to study the impact of a wide array of toxic chemicals and drugs on development, reproduction, and general metabolism.

In this work, we studied the mechanisms through which three molecules from the triazole family: FON, NOL, and one of their last degradation products—1,2,4-Triazole (1,2,4-T)—inhibit hatching in zebrafish. We established that hatching inhibition caused by these molecules is correlated with an impairment of HGC secretion and, specifically in the case of FON, we demonstrated that the effect can be explained by a dopaminergic mechanism that could be part of an endocrine disruption event impacting hatching control in zebrafish. Our results highlight the importance of hatching as a sensitive response to environmental contaminants and drugs that may affect the neuroendocrine system in fish.

## 2. Results

### 2.1. Triazoles Reduce Hatching Success in Zebrafish Embryos

Hatching success was evaluated and recorded in 4 dpf zebrafish; hatching normally occurs at 2 dpf. We tested varying concentrations of FON, NOL, and 1,2,4-T and compared treated animals to untreated sibling controls. Exposure concentrations (Table S1) are equivalent to the calculated LC_50_96h, hatching IC_50_96h, and IC_50_96h/10 for each compound. These values are referred to from now on as high (LC_50_96h), medium (IC_50_96h), and low (IC_50_96h/10) exposure concentrations, respectively, for all compounds studied.

**Figure 1:**
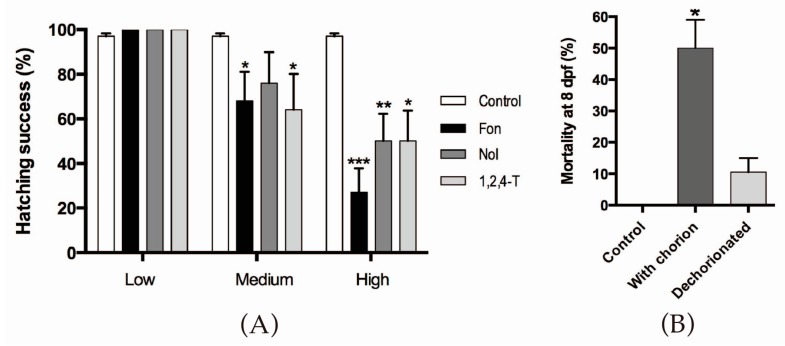
Hatching inhibition in zebrafish after triazole exposure. (**A**) The percentage of hatching success at 4 dpf of larvae exposed to different concentrations of triazoles during embryogenesis is shown. LC_50_96h, hatching IC_50_96h, and IC_50_96h/10 were used as the high, medium, and low exposure concentrations respectively; (**B**) The percentage of dead larvae under each condition at 8 dpf was quantified. Control, unexposed animals. With chorion, high Triadimefon (FON) exposure and animals left in the chorion. Dechorionated, high FON exposure in which chorion was manually removed. Half of the animals die by the eighth day if left in the chorion, but if they are dechorionated at day 2, lethality decreases to about 10%. Exact concentration details are presented in Table S1; (**A**,**B**) Kruskal-Wallis, Dunn’s multiple comparisons test (statistical significance is compared with respective controls * *p* < 0.05, ** *p* < 0.01, *** *p* < 0.001).

Low concentrations of triazoles did not affect the escape from the chorion, while medium and high concentrations of each compound caused a significant inhibition of this behavior (Figure 1A). FON was the compound causing the strongest effect. In this experiment, it was also evident that the hatching impairment was lethal for the embryos (Figure 1B). Most non-hatched embryos are dead by 8 dpf; the lethality is reverted, however, if embryos are manually dechorionated. Since the more robust effect was achieved using high concentrations of triazoles and we wished to explore the mechanisms involved, the rest of the experiments were performed under this condition.

### 2.2. Triazoles Do Not Have a Cytotoxic Effect on the HGCs

Hatching inhibition by triazoles could be explained by the death of the HGCs, that produce the hatching enzyme. To address this question, we performed the TdT-mediated x-dUTP Nick End Labeling (TUNEL) assay in control and exposed embryos at 2 dpf. We did not observe enhanced apoptosis in the hatching gland area after triazole exposures (Figure S1). Hence, hatching inhibition by triazoles is not caused by HGC loss. However, since this result did not allow us to rule out cell type specification alterations or functional perturbation of HGCs, we decided to further evaluate the integrity of this cell type by microscopic analyses.

### 2.3. Triazoles Do Not Reduce HGC Number but Modify Their Morphology

**Figure 2:**
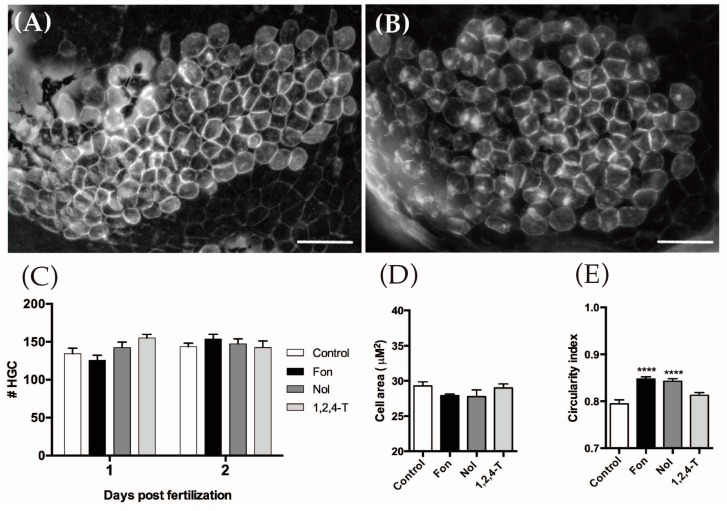
Number and morphology measurements of hatching gland cells (HGCs) in zebrafish embryos exposed to triazoles. (**A**,**B**) Representative images of the pericardial area including the HGCs, labeled with GFP in cldnB:mGFP fish; (**A**) control, and (**B**) representative Triadimefon (FON) and Triadimenol (NOL)-treated 2 dpf embryo; (**C**) There was no difference in the number of HGCs between treated and control embryos at 1 or 2 dpf; (**D**) There was also no difference in cell size at 2 dpf; (**E**) Circularity index measurements were made on HGCs and were compared between treated embryos and controls. The shape of HGCs in treated animals with FON and NOL is more circular compared to untreated ones. Kruskal-Wallis, Dunn’s multiple comparisons test (statistical significance is compared with controls **** *p* < 0.0001). Scale bar: 50 μm.

While we did not detect enhanced cell death in the HGCs by a TUNEL assay, we wondered if a reduction in cell number or abnormal morphology could explain the observed outcome. We analyzed the number, area, and morphology of HGCs in vivo using transgenic cldnB:mGFP embryos [56]. These animals harbor a transgene containing the *cldnB* promoter (*cldnB* is member of the claudin gene family) fused to a gene encoding membrane-bound GFP. Claudins are part of the endothelial/epitheliam tight junction union complex of transmembrane proteins, largely expressed in many tissues on teleost fishes [57], including HGCs [8]. Consistent with the absence of enhanced apoptosis, neither 1 nor 2 dpf-treated embryos showed differences in cell number or cell area with respect to controls. However, HGCs of FON and NOL-treated animals presented a difference in cell morphology compared to control individuals, as they were more circular in shape (Figure 2).

### 2.4. Triazole Exposed Pre-Hatching Embryos Present Reduced Secreted Proteolytic Activity

Since we did not find variations in HGC death or number, but we did see changes in cell form that could be indicative of cytoplasmic content differences, we decided to test whether hatching enzyme release from the HGCs is impaired in triazole-treated fish. We measured protease activity in the perivitelline fluid, where the contents of HGCs are discharged. To isolate perivitelline fluid, we dechorionated embryos in a minimal volume of medium, recovering the resulting solution after different incubation times with triazoles. Protease activity was then measured using the Protease Fluorescente Detection Kit (Sigma) to estimate protease release by comparing it to control standard solutions. We found that, in control embryos, proteolytic activity undergoes a three-fold increase between 46 and 50 hpf, a time that is consistent with hatching onset. In contrast, embryos exposed to high concentrations of triazoles showed only a modest increment in proteolytic activity (Table S2). This result suggests that triazoles are impairing the activity or release of the HE1 enzyme, and likely of other proteins produced by the HGCs.

### 2.5. Triazole-Treated Embryos Retain Granular Content in the Cytoplasm of HGCs

**Figure 3:**
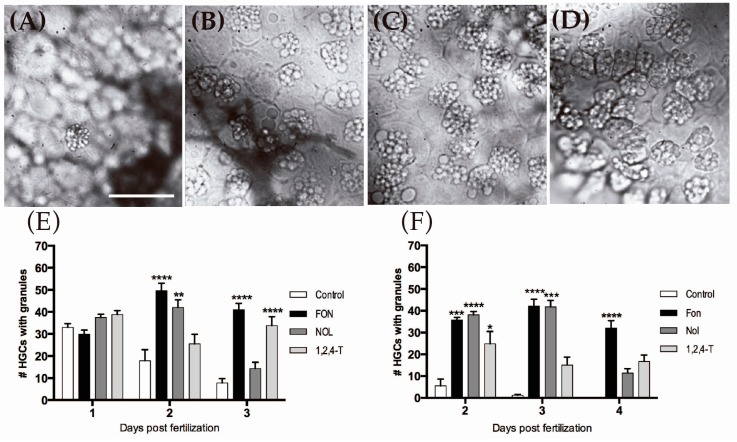
Triazoles prevent secretion of the HGC granules. HGCs with granular content of representative 50–60 hpf (**A**) control, (**B**) FON, (**C**) NOL, and (**D**) 1,2,4-T-treated animals; (**E**,**F**) The number of HGCs containing granules in embryos exposed to a high concentration of triazoles for 48 h starting at blastula (**E**), or 1 dpf (**F**) stages, was counted and averaged. Scale bar: 20 µm. Kruskal-Wallis, Dunn’s multiple comparisons test (statistical significance is compared with controls * *p* < 0.05, ** *p* < 0.01, *** *p* < 0.001, **** *p* < 0.0001).

The decrease in chorion-degrading enzyme HE1 secreted could be due to many factors, including retention of granule contents by HGCs in fish exposed to the triazole compounds. To ascertain if this was the case, we examined HGCs by bright field microscopy and scored for the presence of secretory granules in their cytoplasm (Figure 3). We found that a 48 h exposure to FON and NOL during embryogenesis significantly increased the number of HGCs that retain their granules at 2 dpf (Figure 3E), an effect that is still evident until 3 dpf for FON-treated animals. This finding suggests that triazoles effectively inhibit secretion of the HGC contents, and therefore, the release of the HE1 enzyme into the perivitelline space. We next asked if this was a specific effect on these cells or whether it could be the result of a developmental delay. We carried out an experiment in which exposure was initiated at 24 hpf, instead of at the blastula stage. In this case, granule release was also significantly affected and persisted until 3 dpf for FON and NOL, and 4 dpf for FON (Figure 3F). In all cases, FON again elicited the strongest effect. Therefore, the following experiments aimed at addressing a potential mechanism of action were made only with this agent.

### 2.6. Triadimefon Affects Locomotor Activity in Zebrafish Larvae

**Figure 4:**
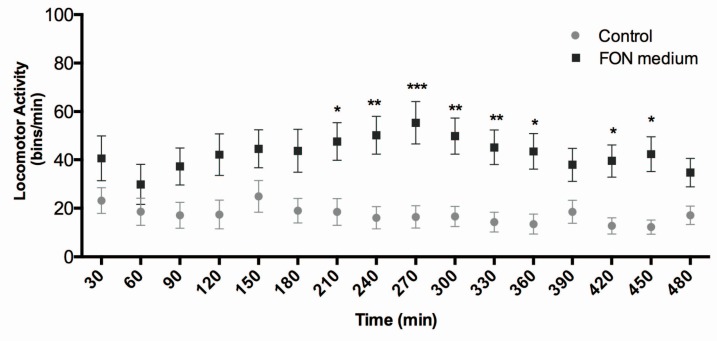
Triadimefon increases locomotor activity (LMA) in 3 dpf larvae. Larvae incubated with the medium FON concentration show a significant increase in LMA when they are compared with non-treated animals. The hyperactivity appears after 3 h of incubation and persists until at least seven hours. Data are presented as average ± SEM from 32 to 40 larvae per condition from four independent experiments. Comparisons were performed using two-way ANOVA, with Sidak’s post-test (statistical significance is compared with controls * *p* < 0.05, ** *p* < 0.01, *** *p* < 0.001).

Previous evidence supports the notion that Dopamine (DA) is a physiological prolactin (PRL)-inhibiting factor of hypothalamic origin in vertebrates [52,53] and that PRL has a stimulating effect on HGC secretion [12]. We thus tested if FON might be interfering with the zebrafish dopaminergic system, an effect that could be reflected by enhanced LMA. We performed an LMA assay in control and FON-treated larvae at 3 dpf using an automated movement recorder. The result shows that the compound induces hyperactivity in the animals after a few hours of treatment, an effect that persisted over many hours (Figure 4). This result suggests that FON may in fact be interfering with dopaminergic signaling in newly hatched zebrafish larvae.

### 2.7. A Dopamine Receptor Antagonist Rescues FON-Induced HGC Granule Release Inhibition

**Figure 5:**
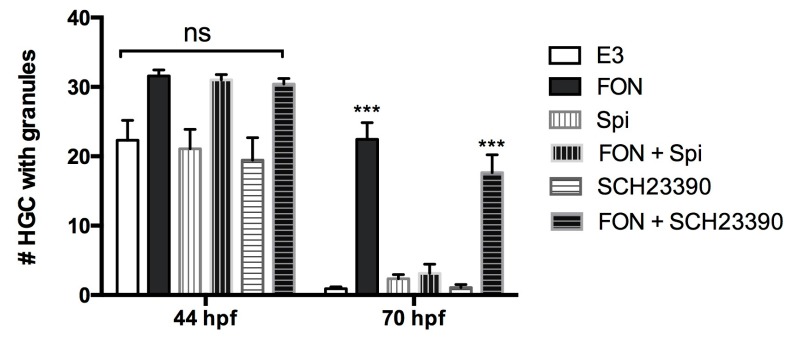
HGC granule secretion is inhibited by FON but rescued by a D2 dopamine receptor antagonist. The graph shows quantification of HGCs with granular content in 44 and 70 hpf larvae under the treatments indicated (E3, control medium; FON, FON high (36 mg/L); incubation was carried out with FON alone, the D2 receptor antagonist Spiperone (Spi), and the D1 receptor antagonist SCH23390 (SCH), or combinations of FON and the antagonists. Granule secretion (naturally occurring arround 48 hpf) is inhibited in fish treated with FON. Coincubation with Spi restores secretion to values indistinguishable from those observed in control conditions; in contrast, SCH coincubation has no significant difference with FON incubation alone. Kruskal-Wallis, Dunn’s multiple comparisons test (statistical significance is compared with respective controls *** *p* < 0.001; ns, not significant).

To further implicate FON as a modulator of the dopaminergic (DA) pathway in zebrafish, and specifically in relationship to hatching, we tested if we could block the effect of the fungicide on HGC granule release with DA receptor antagonists. We tested the effect of two different inhibitors: Spiperone (Spi), an antipsychotic drug that belongs to the butyrophenone chemical class and has been reported to show a high affinity for Dopamine D2 receptors but not for D1 receptors [58,59]; and SCH23390 (SCH), an halobenzazepine known as a potent and selective dopamine D1-like receptor antagonist [60] both in mammals and zebrafish [61]. The inhibitors were incubated alone or in the presence of FON and we determined the number of HGCs containing secretory granules in the fish at two different timepoints (pre- and post-hatching). The result is shown in Figure 5. The D2 receptor antagonist Spi rescued granule secretion to control levels in the presence of FON; in contrast, SCH23390, the D1 receptor antagonist, did not rescue granule secretion.

### 2.8. Acute FON Exposure Induces a Significant Change in the Expression of Prolactin mRNA.

Since our results indicate that D2-like receptors are involved in HE1 release, and the dopaminergic system is associated with PRL signaling, we measured changes in prolactin (*prl*) and tyrosine hydroxylase (*th1*) mRNA levels in FON-treated fish with respect to controls using qPCR. While no statistical difference was observed in the expression of *th1*, there was a modest but significant increase in the expression of *prl* mRNA when fish were treated with a medium concentration of FON. While this result is inconsistent with prolactin being an HE1 stimulator, it is consistent with the fact that DA acts on the lactotroph cells by reducing PRL exocytosis and that the regulatory relationship between DA and PRL is through a “short-loop feedback mechanism” [62].

## 3. Discussion

### 3.1. Triazoles Alter Gene Expression, Metabolism, and Development

Triazole molecules, like FON and NOL, bind to sterol 14-demethylase (CYP51) inhibiting ergosterol biosynthesis in fungi. However, numerous studies have reported on the impact of FON on the vertebrate endocrine system. In rats, it modulates the activity of CYP19 aromatase, which transforms androgens into the corresponding estrogens [36,37] and it induces the expression of the *CYP2B* and *CYP3A* families (which contribute to the metabolism of >60% of drugs in human liver microsomes) in the liver. It also affects the production of testosterone in vivo and in vitro [38]. In the hypothalamic–pituitary–thyroid (HPT) axis, FON induces a decrease of T3 and T4 hormones in rats [39]. Similar results have been reported for fish. Exposure to triazoles in adult medaka fish showed changes in the expression of multiple *CYP* genes related to stereidogenesis, retinoid acid (RA) metabolism-signaling, and general xenobiotic metabolism [63]. Likewise, zebrafish larvae showed differential mRNA expression in the HPT axis in the presence of triazoles [41,42].

These and previously mentioned reports reveal that the toxicity induced by triazoles is linked to at least three different molecular mechanisms: (i) endocrine disruption through steroidogenesis impairment; (ii) teratogenic action mediated by RA signaling modulation; and (iii) behavioral impact through dopaminergic pathway disruption. Therefore, it is evident that triazole fungicides have pleiotropic toxic effects in a wide array of animals, representing a risk to human health and the environment.

### 3.2. HGCs as a Target of Environmental Pollutants

To accomplish hatching, fish embryos release proteases into the extraembryonic (perivitelline) space where they digest the chorion from the inside. HGCs are responsible for this event, producing and releasing the hatching enzymes that, in combination with muscle movements, contribute to the degradation and breaking of this protein matrix. This fact makes this group of cells a possible target for the adverse effect of environmental threats, although the molecular mechanism controlling accumulation and release of their content, as well as their ontogenic history, specification, maturation and death, are thus far poorly understood. As mentioned before, previous studies have explored pollutants that can impact fish embryo hatching by perturbing HGC secretion [24] or affecting HE1 choriolysin activity [64]. However, these studies did not address cell functionality. In the present work, potential molecular participants and pathways implicated in the toxic mechanism were investigated.

### 3.3. FON Reduces HE1 Secretion from HGCs by a Dopaminergic Pathway

Our results indicate that the underlying cause of hatching inhibition is not explained by enhanced death of HGCs. We also excluded the possibility that triazoles could be blocking the development or differentiation of this cell type. However, the data from morphometric analyses led us to hypothesize that the observed change in cell morphology could be related with impaired functionality, possibly involving the secretory function and consequently the release of hatching enzyme, HE1. This was confirmed by the granule release assay (Figure 3) that allowed us to demonstrate that triazoles are capable of blocking the release of the granular content of the HGCs, therefore reducing the liberation of the choriolysin HE1 to the PVS and impairing hatching.

Locomotor stimulation induced by FON exposure (Figure 4) indicates that in fact this molecule could be affecting the CNS of the larvae, possibly via a dopaminergic mechanism, since it is well known that this fungicide can inhibit DA recapture in the synaptic space. On the other hand, we showed that a rescue of HGC secretion reduction induced by FON is possible by coincubation with a DA receptor inhibitor (Figure 5). These results are consistent with a previous report [11] that indicates that DA can indirectly block the release of HE1 from the HGCs, presumably by interfering with PRL secretion. We thus hypothesize that FON is impeding zebrafish embryo hatching through a dopaminergic mechanism that involves the D2-like DA receptors. Nonetheless, we cannot rule out that FON is generating other effects through the D1 receptor pathway. For instance, the hyperactivity and sensibilization produced in rats repeatedly exposed to FON is prevented by SCH and other antagonists of D1 DA receptors [65] and not by blockade of D2 receptors. This is a question that requires further research to be answered and is beyond the scope of this article.

### 3.4. FON Modulates *prl* Expression

**Figure 6:**
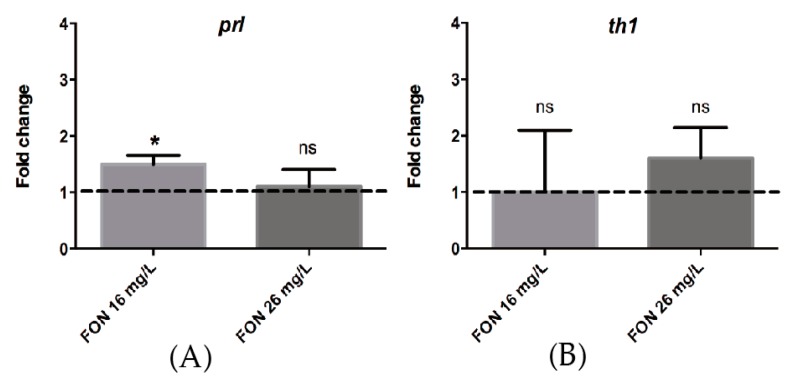
Triadimefon induces the expression of prolactin after 3 h of treatment in 42 hpf embryos. Pfaffl calculated fold-change in embryos incubated with FON at 26 and 16 mg/L relative to non-treated animals. FON 16 mg/L affects prolactin (*prl*) expression (**A**) but does not induce a significant change in tyrosine hydroxylase (*th1*) mRNA levels (**B**). Unpaired *t*-test with Welch’s correction was performed for statistical analysis. * *p* < 0.05; ns, not significant.

Using qPCR analysis, we showed that FON modifies the expression of *prl* mRNA (Figure 6) after a three-hour exposure to 16 mg/L (medium concentration) in 42 hpf zebrafish embryos, just before HGC content release and subsequent hatching. In multiple repetitions of this experiment, using longer time periods or higher concentrations, the expression of three different housekeeping control genes (β-actin, ef1α, gadph), were consistently affected by the FON treatment [66]. Thus, we were unable to use experimental conditions identical to those that elicited the hatching inhibition phenotype. The exposure time is relevant in this case, because the D2-like receptor antagonist Spi, rescued HGC secretion at 72 hpf, a stage were dopamine neurons start to project to the anterior pituitary [67]. This stage would have been the ideal time to measure prolactin expression, but it would require more than 30 h of incubation. Nonetheless, despite appearing counterintuitive, the increase in *prl* expression (Figure 6) is congruent with the reports indicating a stimulating role of *prl* on hatching.

We propose that FON is affecting the secretion of HGCs indirectly by the inhibition of DA re-uptake in the synaptic space and the concurrent increase of DA signaling in the hypothalamus, which, in turn, prevents PRL release from the lactotroph cells in the anterior pituitary, the main mechanism by which DA negatively regulates PRL activity [62]. Another way by which DA modulates PRL action is by decreasing its synthesis, an effect opposite to that of our qPCR results (Figure 6). It is important to note that the regulatory relationship between DA and PRL is highly complex, and that this hormone regulates its own secretion through a feedback mechanism. Moreover, PRL is a very versatile hormone that has more than 300 different biological actions in vertebrates, responding to multiple regulatory mechanisms by many different endogenous and exogenous signals [62]. Thus, our result could be explained by the action of FON through an unknown mechanism, an outcome that can be expected due to the pleiotropic nature of its action and its multiple toxic effects. Finally, our results strongly suggest that the dopaminergic alteration mediated by FON is part of an endocrine disruption mechanism that reduces the secretion of HGCs by impairing the hormonal signal necessary for their proper function at the correct time in order for the embryo to reach the free living stage.

Our results provide the foundation for new studies on the nature and sensitivity of HGCs as a new toxicity target cell type, and for new toxicity bioassay endpoints that can supply information that is of more use than embryonic death alone. In zebrafish, hatching success and locomotor activity can be considered central responses to the presence of environmental pollutants or pharmaceutical compounds. This study further highlights the relevance of using zebrafish as a biological model for toxicology, since our results are consistent with those obtained in mice. Therefore, it reinforces the current trend of using zebrafish based bioassays for performing toxicology and pre-clinical studies.

## 4. Materials and Methods

### 4.1. Animals

Zebrafish were maintained and raised in our facility under recommended conditions [54]. The following strains of fish were used in this study: *TAB5*, a gift of Shawn Burgess) or *tg*(*cldnB:mGFP*), a kind gift of Darren Gilmour [56]. All embryos were collected by natural spawning, staged according to Kimmel et al. [28] and raised at 28 °C in E3 medium (5 mM NaCl, 0.17 mM KCl, 0.33 mM CaCl_2_, 0.33 mM MgSO_4_, equilibrated to pH 7.0) in Petri dishes until treatments begins. Embryonic and larval ages are expressed in hours post-fertilization (hpf) or days post-fertilization (dpf). All animals subjected to experimentation were anesthetized in MS-222 (tricaine; A5040; Sigma, Saint Louis, MO, USA), and procedures compled with the guidelines of the Animal Ethics Committee of the University of Chile.

### 4.2. Chemicals and Determination of Exposure Concentrations

Triazole compounds used as reference toxicants were the antifungals Triadimefon (CAS: 43121-43-3) and Triadimenol (CAS: 55219-65-3), and the final degradation product of this chemical family, 1,2,4-Triazole (CAS: 37306-44-8). To use sub-lethal concentrations that affect hatching, and to achieve exposure conditions that would allow us to study the underlying mechanism, we used the lethal and hatching inhibition concentrations for the 50% of a zebrafish embryo population after a 96 h exposure (LC_50_ and IC_50_), determined by the FET Test method [22], reported previously [68] (Table S1). Since all incubations were made for a maximum of 48 h, the LC_50_ and IC_50_ concentrations never produced significant death in the experimental individuals. The LC_50_, IC_50_, and IC_50_/10 for each compound, are referred to as: high, medium, and low exposure concentrations, respectively, for all compounds studied.

### 4.3. Hatching Success Assessment

To assess the impact of triazoles on zebrafish embryo hatching, groups of 20 TAB5 wild type zebrafish embryos, from 3 to 5 hpf were incubated for 48 h at 26 °C in 6-well multiwell plates with 5 mL of medium at different triazole concentrations using as a negative control E3. Hatching success (number of embryos that escape the chorion) was recorded daily until 4 dpf. In addition, to evaluate the survivability of those embryos that were impeded to hatch by 4 dpf, we manually dechorinated a group of them and compared their survival with the non-hatched embryos until 8 dpf.

### 4.4. Cell Death Detection

To assess apoptotic cell death in the HGCs, TUNEL (TdT- mediated x-dUTP Nick End Labeling) was performed using the In Situ Cell Death Detection Kit TMR Red (Roche, Mannheim, Germany), following the manufacturer’s instructions.

### 4.5. Counting and Measurement of HGCs

For counting and morphological measurements of HGCs, incubations were performed as described above, but embryos were manually dechorionated just before evaluation between 1 and 2 dpf. We used the *tg*(*cldnB:mGFP*) [56] transgenic line for these experiments because we serendipitously found that these animals display strong membrane bound GFP expression in HGCs. Nonetheless, HGCs can also be easily identified by their granular content, size, position, and transient nature, thus allowing clear visualization and imaging of these cells (Figure 3). Under a fluorescent stereoscope (Olympus, MVX10, Tokyo, Japan) connected to a digital camera (QImaging^®^, Surrey, BC, Canada) photographs were taken of the HGCs on one side of each individual. GFP positive cells on the pericardial surface of the egg yolk were counted and measured. For cell measurements, 70 cells from 10 embryos were analyzed for each treatment (7 cells/embryo). For cell counting, 11 to 25 individuals were analyzed per treatment and stage. Images were processed and analyzed using Adobe Photoshop CS4. Results were later statistically analyzed with Student’s *t*-test using the software Graph Pad Prism version 6 for OSX (GraphPad Software, San Diego, CA, USA).

### 4.6. Proteolytic Enzyme Secretion Assay

Protease release from the embryos at hatching time was evaluated by incubating groups of 20 dechorionated embryos of 42 hpf for up to 8 h in E3 or E3 with high concentrations of each compound. Embryos were kept individually in 96-multiwell plates with 100 μL of medium (E3 or treatment). After 4 or 8 h, 80 μL of medium was extracted from each well, collected, pooled, and kept on ice. Samples were centrifuged at 18,000× *g* for 30 min at 4 °C and concentrated 50 times using Amicon 10 K Microfilters Ultra 0.5 Millipore (Merk Darmstadt, Germany). For proteolytic activity evaluation, the Protease Fluorescent Detection Kit (Sigma) was used following the manufacturer’s instructions in a Luminiscence Spectrometer LS50 (Perkin Elmer, Waltham, MA, USA). Proteolytic activity was expressed as arbitrary units of fluoresce emission at 525 nm.

### 4.7. Quantification of HGCs with Granular Content

To evaluate the secretory activity of HGCs, the number of cells in a predefined area, containing granules in the cytoplasm, was quantified daily between 1 and 4 dpf in embryos exposed for 48 h from blastula stage, or for 24 hpf, to high concentrations of all triazoles. For secretion rescue experiments, embryos of 30 hpf were incubated in 5 mL of FON or co-incubated with dopamine receptor antagonists and their respective controls. The D2-like receptor antagonist Spiperone (Spi) was used at 1 μM in 0.02% DMSO as a vehicle and the D1-like receptor antagonist SCH 23390 (SCH) at 2 μM. Incubation took place for 12 h at 28 °C in darkness. HGCs were imaged by Bright field microscopy (Carl Zeiss optical microscope) and the number of cells—present in a predefined area—which retained their granular content, was determined at different times before and after hatching. Approximately 20 individuals for each treatment and stage were analyzed and the experiment was repeated at least three times.

### 4.8. Quantitative PCR

Quantitative qPCR was performed to determine changes in the mRNA expression levels of the prolactin (*prl*) and tyrosine hydroxylase (*th1*) genes. Embryos (*n* = 25–50) were incubated at 39 hpf with FON at 16 and 26 mg/L. Total RNA was collected at 42 hpf using TRIzol Reagent (Life Technologies, Carlsbad, CA, USA). The cDNA was prepared from 1 µg total RNA with oligo (dT) primer in a 20 µL reaction volume using Improm-II Reverse Transcription System (Promega, Madison, WI, USA) and finally diluted in 40 µL of water. Real time PCR was set up using 2 µL cDNA, 10 µL SYBR Green Master Mix (Agilent Technologies, West Cedar creek, TX, USA) and 250 nM of each forward and reverse primers in a total volume of 20 µL. The qPCR was run for 40 cycles in a Stratagene Mx3000P thermocycler (Agilent Technologies, Waldbronn, Germany). Primers were obtained from Chen et al. [69] for *th1* (Forward 5′-GACGGAAGATGATCGGAGACA-3′, Reverse 5′-CCGCCATGTTCCGATTTCT-3′) and from Hoshijima et al. [70] for *prl* (Forward 5′-GGCCTGGAGCACGTCGTA-3′, Reverse 5′-ACGGGAGAGTGGACAGGTTGT-3′). We used *gadph* mRNA levels for normalization, and the relative quantification of gene expression was calculated using the Pfaffl method. The data was displayed as a fold difference in experimental animals relative to non-treated embryos. The resulting value in each condition was calculated from three independent experiments. Statistical significance was determined by unpaired two-tailed Student’s *t*-test using Welch’s correction with *p* < 0.05.

### 4.9. Locomotor Activity Quantification

To assay locomotor activity, 3 dpf larvae were incubated in a 96-well multiwell plate with 250 µL of FON 16 mg/L at 28 °C during 8 of light. Locomotor activity measurements were provided by the automated system Microtracker [71]. The fungicide was added to media just before recording, therefore, recording and incubation time are equivalent in the graph. Statistical analyses were performed using GraphPad Prism version 6 for Windows software (GraphPad Software).

## Supplementary Materials

Supplementary materials can be found at www.mdpi.com/1422-0067/18/4/710/s1.

## Abbreviations

1,2,4-T	1,2,4-Triazole
DA	Dopamine
FON	Triadimefon
GFP	Green fluorescent protein
HE1	Hatching enzyme 1
HGC	Hatching gland cells
hpf	Hours post-fertilization
HPT	Hypothalamic-pituitary-thyroid
IC_50_	Inhibition concentration 50
LC_50_	Lethal concentration 50
LMA	Locomotor Activity
NOL	Triadimenol
PRL	Prolactin
PVS	Perivitelline space
SPI	Spiperone
SCH	SCH23390
