# Supplementary material for: Triazole Fungicides Inhibit Zebrafish Hatching by Blocking the Secretory Function of Hatching Gland Cells
Source: Int J Mol Sci. 2017 Apr 4;18(4):710. doi: 10.3390/ijms18040710 (PMC5412296; doi:10.3390/ijms18040710)
Supplement: Supplementary file 1 [file ijms-18-00710-s001.pdf]

# Supplementary Materials: Triazole Fungicides Inhibit Zebrafish Hatching by Blocking the Secretory Function of Hatching Gland Cells

**Javiera F. De la Paz, Natalia Beiza, Susana Paredes-Zúñiga, Misque Hoare and Miguel L. Allende**

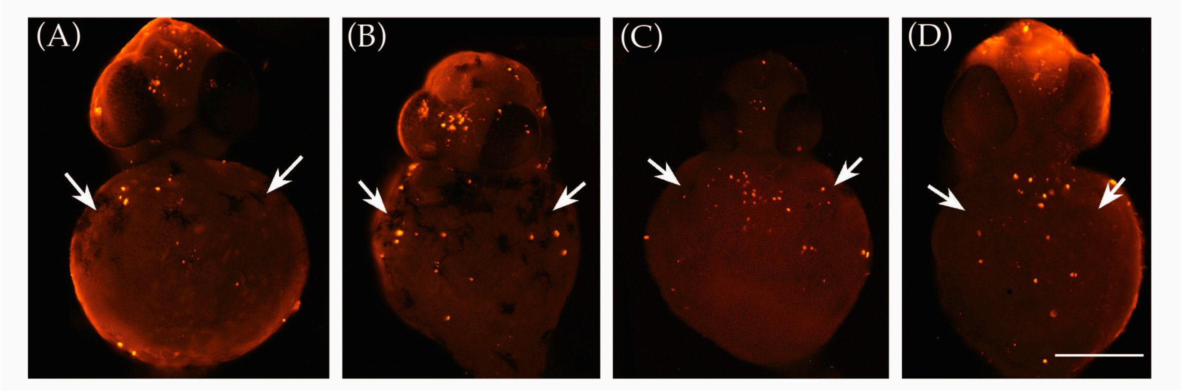


**Figure S1.** Apoptosis in not activated by triazole exposure. Representative images (ventral views) of 2 dpf embryos. (**A**) control untreated; (**B**–**D**) embryos exposed to high concentrations of (**B**) FON, (**C**) NOL and (**D**) 1,2,4-T. White arrows indicate hatching gland area. Scale bar: 200 µm.

**Table S1.** Triazole concentrations used for treatments. Mean lethal ($LC_{50}$) and hatching inhibition concentrations ($IC_{50}$) after 96 h were determined by the FET Test. $LC_{50}$96h, $IC_{50}$96h and $IC_{50}$96h/10 were used as high, medium and low exposure concentrations, respectively.

| Compound | $LC_{50}$96h | $IC_{50}$96h | $IC_{50}$96h/10 |
|---|---|---|---|
| FON (mg/L) | 36.4 | 16.3 | 1.6 |
| NOL (mg/L) | 55.5 | 28 | 2.8 |
| 1,2,4-Triazole (g/L) | 12 | 5.6 | 0.6 |
| Concentration category | High | Medium | Low |

**Table S2.** Protease release at the hatching stage. * Proteolytic activity was measured in the extraembryonic medium of treated and control embryos at two stages, pre hatching (46 hpf) and hatching (50 hpf), as indicated. While in control embryos, the activity more than doubles between pre-hatching to hatching stages, treated animals only modestly enhance or reduce their proteolytic activity release at hatching stage. Fluorescence emission at 46 and 50 hpf on control and treated dechorionated embryos. E3 was measured as a blank. hpf, hours post fertilization.

| Sample | Control | FON | NOL | 1,2,4-T | E3 | Trypsin |
|---|---|---|---|---|---|---|
| 46 hpf | 230 | 230 | 204 | 170 | 149 | 425 |
| 50 hpf | 553 | 309 | 227 | 159 | 149 | 425 |

* Proteolitic activity estimated as arbitrary units of fluorescence emission at 535 nm.
